# Preparation and Surface Characterization of Cerium Dioxide for Separation of ^68^Ge/^68^Ga and Other Medicinal Radionuclides

**DOI:** 10.3390/ma16051758

**Published:** 2023-02-21

**Authors:** Kateřina Ondrák Fialová, Kryštof Adámek, Martin Vlk, Barbora Drtinová, Karel Štamberg, Ferdinand Šebesta, Miroslav Šlouf, Ján Kozempel

**Affiliations:** 1 Faculty of Nuclear Sciences and Physical Engineering, Czech Technical University in Prague, Břehová 7, 115 19 Prague, Czech Republic; 2 Institute of Macromolecular Chemistry, Czech Academy of Sciences, Heyrovského náměstí 2, 162 06 Prague, Czech Republic

**Keywords:** cerium dioxide, XRPD, FT-IR, SEM, TEM, thermal analysis, acid-base titration, sorption, mechanism

## Abstract

The overall need for the preparation of new medicinal radionuclides has led to the fast development of new sorption materials, extraction agents, and separation methods. Inorganic ion exchangers, mainly hydrous oxides, are the most widely used materials for the separation of medicinal radionuclides. One of the materials that has been studied for a long time is cerium dioxide, a competitive sorption material for the broadly used titanium dioxide. In this study, cerium dioxide was prepared through calcination of ceric nitrate and fully characterized using X-ray powder diffraction (XRPD), infrared spectrometry (FT-IR), scanning and transmission electron microscopy (SEM and TEM), thermogravimetric and differential thermal analysis (TG and DTA), dynamic light scattering (DLS), and analysis of surface area. In order to estimate the sorption mechanism and capacity of the prepared material, characterization of surface functional groups was carried out using acid-base titration and mathematical modeling. Subsequently, the sorption capacity of the prepared material for germanium was measured. It can be stated that the prepared material is prone to exchange anionic species in a wider range of pH than titanium dioxide. This characteristic makes the material superior as a matrix in ^68^Ge/^68^Ga radionuclide generators, and its suitability should be further studied in batch, kinetic, and column experiments.

## 1. Introduction

Ion exchange is by far the most natural and versatile separation method benefiting from the large capacity of ion exchangers, fast kinetics of separation processes, and a quite simple recovery of separated ions [1].

Even though organic resins still belong to the most common and widely available ion exchangers because of their higher capacity and chemical stability, certain properties make them inferior to inorganic ion exchangers, mainly in nuclear applications where high selectivity and radiation stability are crucial. Lower radiation stability leads to lowering the capacity of organic material and, together with low selectivity, to lowering the radionuclide purity of the final sample. Radiation degradation of organic material also lowers the final chemical purity, which is particularly crucial in the radiopharmaceutical industry [2].

Inorganic ion exchangers, on the other hand, can be tailored to fit the exact separation process. They can operate under extreme thermal or chemical conditions. This makes them a perfect material for use in ion exchange chromatography. They also possess superior radiation stability, so their use in chromatography does not have to be limited to a single-cycle process. Thus, they can be conveniently used as active components in radionuclide generators, devices used for repeated recovery of daughter radionuclides, while the mother radionuclide is permanently anchored to the chromatographic column [1,3].

Even though there are a variety of natural inorganic ion exchangers, synthesis is used to design and tailor a material for a specific purpose. Synthetic inorganic ion exchangers can be classified as:

1. Synthetic zeolites
2. Heteropolyacids
3. Metal ferrocyanides
4. Polybasic acid salts
5. Insoluble ion exchange materials (e.g., sulphides)
6. Hydrous oxides

In radiopharmacy, hydrous oxides belong to the most studied group of synthetic inorganic ion exchangers. Trivalent and tetravalent metal oxides have already found multiple uses in the separation of various medicinal radionuclides. They owe this broad area of use mainly to their amphoteric character:

M–OH ↔ M^+^ + OH^−^ pH < 7 (1)

M–OH ↔ M–O^−^ + H^+^ pH ˃ 7 (2)

In an acidic medium, they undergo the dissociation, as indicated in Equation (1) and therefore exchange anionic species. In a basic medium, they act as cation exchangers, as indicated in Equation (2) [4].

The main advantage of using these materials in the radiopharmaceutical industry is the possibility of radionuclide recovery in an ionic form instead of in an organic complex, which facilitates further use of the radionuclide [5,6]. In practice, mainly aluminum oxide [7], stannous dioxide [8], zirconium dioxide [9], or titanium dioxide [10] are sorbents of choice. For example, titanium dioxide is used in the solid phase in ^68^Ge/^68^Ga radionuclide generators, which are eluted by 0.1 M hydrochloric acid, with an elution yield of ^68^Ga around 70% [5,6,11].

Another oxidic material with possible use for separation of ^68^Ga is cerium dioxide, mainly in its nanoparticulate form [12,13]. For separation of ^68^Ga, this material was introduced in the work of Bao and Song [12] and further studied by Chakravarty [13]. In the former work, cerium dioxide prepared by thermal decomposition of nitrates was used to anchor ^68^Ge and 0.02 M HCl to elute ^68^Ga in a yield of 56% [12]. Chakravarty used nanoceria-PAN, a composite of cerium dioxide nanoparticles prepared by calcination of cerium oxalate and polyacrylonitrile (PAN), and 0.01 M hydrochloric acid to obtain around 80% yield of ^68^Ga. The use of a composite of nanoparticles and a polymer matrix seems to be beneficial for the chemical and mechanical stability of the material. Furthermore, a significant decrease in the molarity of the acid used compared to titanium dioxide would be beneficial for further use of the ^68^Ga eluate [6]. Further elaboration and study of this approach are desirable.

Apart from that, cerium dioxide is used and studied in electrochemistry, fuel or solar cells, and medicine, for example, as an antioxidant enzyme mimicker or imaging pharmaceutical agent [14,15,16,17,18]. Cerium dioxide is often used as a solid electrolyte thanks to its high conductivity and low activation energy. It can operate at relatively low temperatures and is used in solid oxide fuel cells as either an electrolyte or a catalyst [18]. Its redox and catalytic properties make it an indispensable part of automotive three-way catalysts [19]. In photovoltaics, cerium oxide improves the UV and thermal stability of solar cells thanks to its broad bandgap. In the form of nanoparticles, it also exhibits photoactive properties. Moreover, its absorption spectrum is shifted to the visible region of the solar spectrum compared to that of other commonly used materials, such as titanium dioxide [15]. It has been used, for example, in perovskite solar cells as part of an electron transport layer [20,21]. In medicine, cerium dioxide is used to mimic the activity of various enzymes [17]. Its unique redox properties can be used to modulate levels of free radicals and cerium dioxide, and they can also be used either to protect against oxidative stress, which can be mainly beneficial in the case of chronic inflammation or radioprotection [22,23], or to sensitize tumor cells [24]. Moreover, as a metal oxide, it can be used for reflectance imaging [24]. Recently, cerium dioxide has also been studied as a potential radionuclide carrier in combined radiopharmaceuticals [25].

There are diverse methods to prepare cerium dioxide with the desired parameters. Among the traditional approaches for cerium dioxide preparation is the simple calcination of ceric or cerium salts, such as ceric or cerium nitrate, cerium ammonium nitrate [12], or cerium oxalate [13]. Hydrous cerium dioxide can then be prepared by basic hydrolysis of cerium salts (precipitation), mainly ceric or cerium nitrate [26,27] or cerium sulphate [28]. The sol-gel method can also be the method of choice [14]. Solvothermal methods, where water or alcohol are used as a solvent, can also be used to prepare cerium dioxide from cerium ammonium nitrate or cerium acetate [29,30]. Spray pyrolysis can be a method of choice as well [31]. Different methods of preparation and conditions of synthesis lead to differences in the size, shape, and final composition of the oxide/hydrous oxide nanoparticles, and their surface characterization. The calcination method usually produces larger particles in comparison with precipitation or sol-gel methods. This is crucial, especially for medical applications in drug delivery systems [17].

Considering the broad spectrum of use, detailed knowledge of cerium dioxide sorption properties has become very important in many research and industrial fields for both intentional sorption/desorption processes and spontaneous sorption of potential impurities. Sorption mechanisms are particularly important in the separation process. In the case of the ^68^Ge and ^68^Ga pair, it is assumed that germanium in the form of negatively charged species, such as [GeO(OH)_3_]^−^, [GeO_2_(OH)_2_]^2−^, and [[Ge(OH)_4_]_8_(OH)_3_]^3−^, predominate in the system and interact electrostatically with the cerium dioxide surface, which should be positively charged according to Equation (1). Gallium, on the other hand, exists mainly in the form of Ga^3+^ under the chosen conditions, which results in very little (not zero) affinity to cerium dioxide [13]. Mainly the non-zero affinity of positively charged ions and the decrease in necessary elution acid molarity when transferring from titanium dioxide to cerium dioxide indicate the complexity of the sorption mechanism.

As described in [32], the surface of sorption materials usually contains two types of active sites, called edge sites and layer sites. During the change in pH, protonation and deprotonation reactions take place on the edge sites (Equations (3) and (4)) and ion exchange reactions on the layer sites (Equation (5)):

SO^−^ + H^+^ ↔ SOH (3)

SOH + H^+^ ↔ SOH_2_^+^ (4)

XNa + H^+^ ↔ XH + Na^+^ (5)

where SO^−^, SOH, and SOH_2_^+^ are symbols of different forms of edge sites, and XNa and XH are symbols of different forms of layer sites. The surface charge of the material as well as the total concentration of the sites and molar fractions of site formations at certain pH indicate the protolytic properties of the material and might predict the mechanism involved in the sorption processes. This characterization can be carried out using acid-base titration, surface area measurement, and mathematical modeling [32].

In this work, cerium dioxide was prepared by calcination of ceric nitrate hexahydrate and characterized using infrared spectrometry (FT-IR), X-ray powder diffraction (XRPD), thermogravimetric and differential thermal analysis (TG and DTA), scanning and transmission electron microscopy (SEM and TEM), and dynamic light scattering (DLS). The characterization of the surface functional groups was provided in order to estimate the sorption mechanism and capacity of the prepared cerium dioxide from its protolytic properties. As similar characteristics of titanium dioxide are already available in the work of Kukleva et al. [33], the results obtained were compared. The work of Vlasova and Markitan [34] also offers a view of the protolytic behavior cerium dioxide and can be used as a comparative sample.

Subsequently, the sorption capacity of the prepared material for germanium was measured to confirm its applicability in a potential radionuclide generator construction. For that, a composite material of CeO_2_ and polyacrylonitrile (PAN) was prepared.

## 2. Materials and Methods

### 2.1. Synthesis

Cerium dioxide was prepared by calcination of ceric nitrate hexahydrate (99.99%; Sigma-Aldrich, St. Louis, MO, USA) in the vacuum furnace 0415 VAK (Clasic, Řevnice, Czech Republic) at 400 °C for 2 h in a ceramic crucible covered with a loose ceramic cap. After calcination, the sample was cooled in air, and after cooling, the material was crushed in agate mortar and stored.

### 2.2. Characterization

Infrared spectrometry was performed on an FT-IR spectrometer (NICOLET iS50, Thermo Scientific, Waltham, MA, USA) by the attenuated total reflectance technique on a diamond crystal. The spectrum was acquired in the mid-infrared region (400–4000 cm^−1^; resolution: 2 cm^−1^) and subsequently processed with SW OMNIC 9 (Thermo Scientific).

For XRPD analysis, a diffractometer (Rigaku MiniFlex 600–40 kV, 40 mA; Rigaku, Tokyo, Japan) in the range of 10–80° in ϴ–2ϴ geometry with a Cu-Kα1,2 X-ray tube was used. The identification of the crystal phase was carried out using the ICDD PDF-2 database [35]. The crystallite size was determined from the integral width of peaks using the Halder-Wagner method.

Thermogravimetric and differential thermal analysis was carried out using a thermogravimeter (Labsys Evo, Setaram, Caluire-et-Cuire, France) with a crucible holder for 1200 °C and a corundum crucible. The temperature rate was 4 °C/min up to 600 °C, followed by cooling.

The overall morphology, size, composition, and crystal structure of CeO_2_ nanoparticles were analyzed using a high-resolution field-emission gun scanning electron microscope (FEGSEM; microscope MAIA3, Tescan, Brno, Czech Republic) and a transmission electron microscope (TEM; microscope Tecnai G2 Spirit Twin, Brno, Czech Republic) as described elsewhere [36]. Briefly, the nanoparticles were deposited on a standard carbon-coated copper grid and characterized by the following FEGSEM and TEM modes:

1. The secondary electron imaging (FEGSEM/SE) displayed the overall morphology.
2. The bright field imaging (TEM/BF) at 120 kV showed the size and shape of individual nanocrystals.
3. The energy-dispersive analysis of X-rays (TEM/EDX) verified elemental composition.
4. The selected area electron diffraction (TEM/SAED) confirmed the expected crystal structure of CeO_2_ nanoparticles. The experimental electron diffraction patterns were processed with ProcessDiffraction [37] and compared with the theoretically calculated patterns of CeO_2_ using the Python package EDIFF [38] (the cubic modification of CeO_2_ for EDIFF calculation came from the Crystallography Open Database [39]).

For dynamic light scattering analysis, the Zetasizer NanoZS instrument, model ZEN3600 (Malvern Instruments, Malvern, United Kingdom), was used and the intensity-weighted hydrodynamic radius, *R*_H_, scattering intensity, and zeta potential, ζ, were measured. Repeated measurements at 25 °C with a scattering angle of 173° were carried out. The concentration of cerium dioxide was 0.1 mg/mL in ultrapure water.

### 2.3. Characterization of Surface Functional Groups

For analysis of surface functional groups, measurement of specific surface area and acid-base potentiometric titration were performed. The specific surface area of the prepared sample was measured using Monosorb MS-22 (Quantachrome Instruments, Boyton Beach, FL, USA) for fast determination by sorption of the He and N_2_ mixture at −196 °C, followed by desorption at elevated temperature. Potentiometric titration was carried out in the range of pH = 3–11 using a TitraLab 845 titration workstation (Radiometer analytical, Villeurbanne, France) with a 0.05 mL increment of titrant and 20 mpH/min stability. The prepared material (100 mg) was dispersed in 100 mL of 0.1 M NaCl (Sigma-Aldrich) to ensure solution ionic strength. To obtain the acidic branch of the titration curve (pH = 3–5), 1 M HCl (Sigma-Aldrich) was used. In the case of the basic part (pH = 5–11), 0.1 M NaOH (Sigma-Aldrich) was utilized. Basic titration was performed under an inert atmosphere of N_2_.

Using these experimental data and values of specific surface area of the tested material, a mathematical model-based description of protolytic processes occurring in the system with the prepared cerium dioxide was provided. The mathematical models derive from the following assumptions: the surface of sorption materials usually contains two types of surface groups, edge sites and layer sites, where reactions (3)–(5) take place during titration.

Equilibrium constants for reactions (3)–(5) are formulated by Equations (6)–(8):

K_1_ = [SOH]/([SO^−^]∙[H^+^]) (6)

K_2_ = [SOH_2_^+^]/([SOH]∙[H^+^]) (7)

K_ex_ = ([XNa]∙[H^+^])/([XH]∙[Na^+^]) (8)

For total concentration as well as charge densities of edge sites and layer sites, Equations (9) and (10) apply:

∑SOH = [SOH] + [SO^−^] + [SOH_2_^+^] (9)

∑X = [XH] + [X^-^] ≅ [XH] + [XNa] (10)

For description of processes taking place on edge sites, one of the following surface complexation models (SCMs) is used: constant capacitance model (CCM), diffusion double-layer model (DLM), or non-electrostatic chemical equilibrium model (CEM). The ion exchange model describes (IExM) processes taking place on layer sites. A detailed description of these models is given in [32].

The mentioned models can be further applied assuming that the concentration of the ith component in the aqueous layer adhering to the surface (*C*_i_)_s_ is given by the Boltzman Equation (11):

(*C*_i_)_s_ = *C*_i_∙exp(−*zψF*/*RT*) (11)

where *C*_i_ is a bulk concentration of the *i*th component, *z* is its charge, *ψ* is the electrostatic potential, *F* is the Faraday constant, *R* is the gas constant, and *T* is the absolute temperature [32].

The relationship of surface charge σ and electrostatic potential *ψ* is then described for the named models CCM, DLM, and CEM, respectively, as:

*σ* = *G*∙*ψ* (12)

*σ* = 0.1174∙*I*^1/2^∙sinh(*zψF*/2*RT*) (13)

*ψ* = 0 (14)

where *I* is ionic strength and *G* is the so-called Helmholtz capacitance [32].

The above-described models were applied to experimental data using the software FAMULUS (code package: STAMB 2015; code: P46DNRLG.fm), including the Newton-Raphson multidimensional nonlinear regression method. The suitability of a model for description of experimental data was evaluated by the WSOS/DF value (weighted sum of squares of differences divided by degrees of freedom) as a criterion of goodness-of-fit. To accept a model, the WSOS/DF value should be in the range 0.1–20—the lower the better—taking into consideration values of all constants and total concentrations of sites derived from the model used.

### 2.4. Measurement of Sorption Capacity for Germanium

In order to measure the sorption capacity of the prepared material, CeO_2_ was granulated into a polyacrylonitrile (PAN) matrix, as pure powder material would not allow direct application in column experiments. The composite material was prepared using an already published method by coagulation of PAN and CeO_2_ reaction mixture in a water bath [40]. First, the suspension of CeO_2_ in the solution of PAN in dimethyl sulfoxide was prepared so the ratio of active agent in the composite would be 85% in dry state. The mixture was dispersed through a dedicated apparatus into a water bath. The beads formed were sieved, and particles with the size range 0.3–0.6 mm were used in the experiment. The final composite contained 222 mg of CeO_2_ per 1 mL of wet material.

For the measurement of sorption capacity for germanium, a column packed with 1.25 mL of the prepared composite was used. The stock solution of 0.1 M hydrochloric acid contained 4.08 mol/L of germanium chloride (AlphaAesar, Haverhill, MS, USA) and was spiked by 50 μL of ^68^Ge solution in 0.5 M hydrochloric acid (Eckert & Ziegler, Berlin, germany; 14.8 MBq/mL). The stock solution was pumped through the packed column via the peristaltic pump PDC 22 (Čerpadla Kouřil, Kyjov, Czech Republic) at a rate of 4 mL/h. A fraction of approximately 4 mL was collected and weighted in order to determine the precise volume. Aliquots of 1 mL were measured after 24 h using the NaI(Tl) well detector NKG 314 (Tesla, Prague, Czech Republic). The breakthrough curve was constructed as the dependance of the *A*/*A*_0_ ratio, where *A* is the activity in the sample aliquot and *A*_0_ is the input activity of 1 mL aliquot, on volume *V*. The total capacity of CeO_2_ for germanium up to *A*/*A*_0_ = 1 was calculated considering the dead volume of the column, *V*_0_ = 0.6 mL, from the surface above the breakthrough curve.

## 3. Results and Discussion

### 3.1. Characterization of Prepared Cerium Dioxide

**Figure 1:**
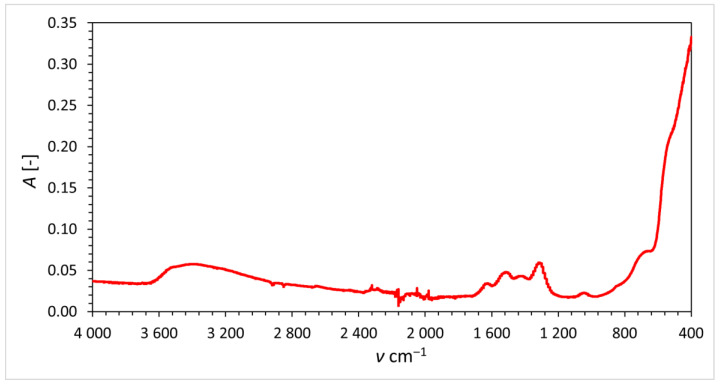
FT-IR spectrum of the prepared cerium dioxide.

The FT-IR spectrum of the prepared material is depicted in Figure 1. The small, broad band at around 3450 cm^−1^ and the small band around 1600 cm^−1^ can be attributed to O-H stretching and deformation vibrations of crystalline water. The intense band around 500 cm^−1^ belongs to Ce-O stretching vibration. The small band around 850 cm^−1^ indicates metal-oxygen stretching vibration, which corresponds to metal impurities in ceric nitrate hexahydrate.

**Figure 2:**
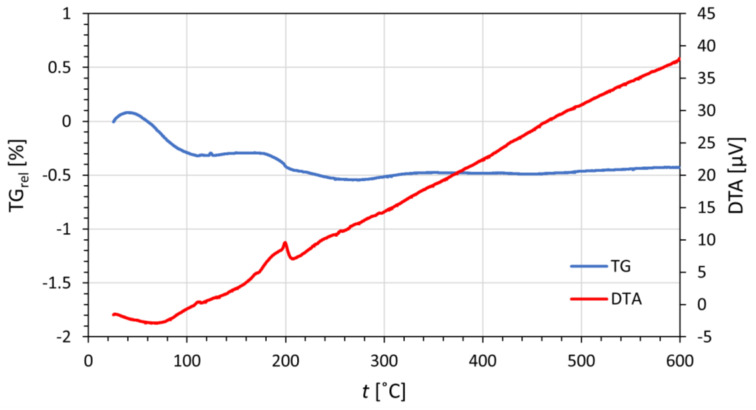
Thermogravimetric and differential thermal analysis; dependence of relative weight loss TG_rel_ and heatflow on furnace temperature.

The presence of water residue was also confirmed by TG and DTA analysis, as seen in Figure 2. The relative mass losses up to 0.5% around 100 and 200 °C indicate the loss of a small quantity of atmospheric and crystal water contained in the sample. The water residue probably comes from readsorption of water on the surface of cerium dioxide, a phenomenon common to all oxidic materials.

**Figure 3:**
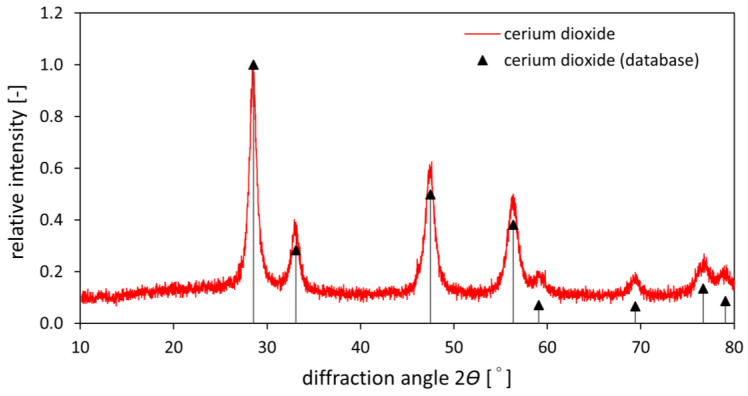
XRPD of the prepared cerium dioxide with database data for comparison.

The prepared material was further studied using XRPD. The diffractogram is shown in Figure 3, together with database data. It can be seen that the measured diffractogram corresponds well with the database data of cerium dioxide. From the integral width of the peaks, the crystallite size was estimated to be 10.8 ± 0.3 nm.

**Figure 4:**
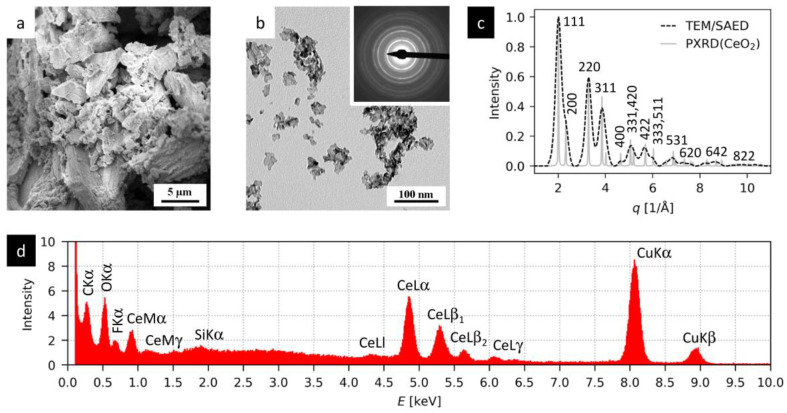
FEGSEM and TEM analysis of the prepared nanocrystals: (**a**) FEGSEM/SE micrograph showing a continuous layer of nanocrystals and their agglomerates deposited on a microscopic glass, (**b**) TEM/BF micrograph showing morphology of individual nanocrystals deposited on electron-transparent carbon film (inset: powder TEM/SAED diffraction pattern), (**c**) comparison of the experimental TEM/SAED pattern with theoretically calculated X-ray diffraction pattern (PXRD) corresponding to the cubic modification of CeO_2_, and (**d**) energy-dispersive spectrum (TEM/EDX) of the nanocrystals.

Figure 4 summarizes the results of the electron microscopy analysis of the prepared CeO_2_ particles. The CeO_2_ crystals exhibit quite a broad size distribution, as evidenced in the FEGSEM/SE micrograph (Figure 4a), where some agglomerates are >1 μm, and the TEM/BF micrograph (Figure 4b), where some crystals are <10 nm. The TEM/SAED diffraction pattern (inset in Figure 4b) documents the nanocrystalline character of the prepared nanoparticles. The comparison of the radially averaged experimental TEM/SAED pattern with the EDIFF-calculated powder X-ray diffraction pattern (PXRD) of CeO_2_ (Figure 4c) verifies that the nanoparticles exhibit the cubic crystalline modification of cerium dioxide. TEM/EDX analysis (Figure 4d) confirms the elemental composition of CeO_2_: all strong peaks in the EDX spectrum correspond to cerium dioxide (Ce, O) or to the supporting carbon-coated copper grid (C, Cu). The low-intensity fluorine peak at 0.67 keV can be attributed to a residual impurity, while the small silicon peak at 1.74 keV can be interpreted as a fluorescence from the Si detector of the EDX system [41].

Using DLS analysis, it was determined that one broad peak occurs in the distribution diagram (see supplementary information Figures S1 and S2). With repeated measurements, the volume mean radius was determined to be 487 ± 71 nm. However, the particles are almost electrically neutral, with *ζ* potential of 0.52 ± 0.25 mV, which signifies the low stability of the colloidal system and the vast agglomeration of the prepared particles in water. This means that the measured value of the particle size does not necessarily agree with the real state. These findings are not surprising, as cerium dioxide nanoparticles were prepared in dry state and were not intended for use as part of any colloidal system.

From the results described in Section 3.1, it can be stated that the chosen method of synthesis, calcination of ceric nitrate at 400 °C, is suitable for the preparation of cerium dioxide. The performed analyses confirmed that crystalline cerium dioxide was prepared using the chosen method. The particle size range was determined by microscopy as quite broad, from around 10 nm to 1 μm. DLS analysis confirmed the electrical neutrality of the prepared particles, explaining the agglomeration of particles in the water phase. These characteristics do not interfere with the intended use of the prepared material in the separation of medicinal radionuclides, as a similar approach as in [13] will be adopted for further use of the prepared material. It will be granulated in an inert polymer matrix to improve the granulometric, mechanical, and physical qualities for use in radionuclide generators.

### 3.2. Characterization of Cerium Dioxide Surface Functional Groups

**Figure 5:**
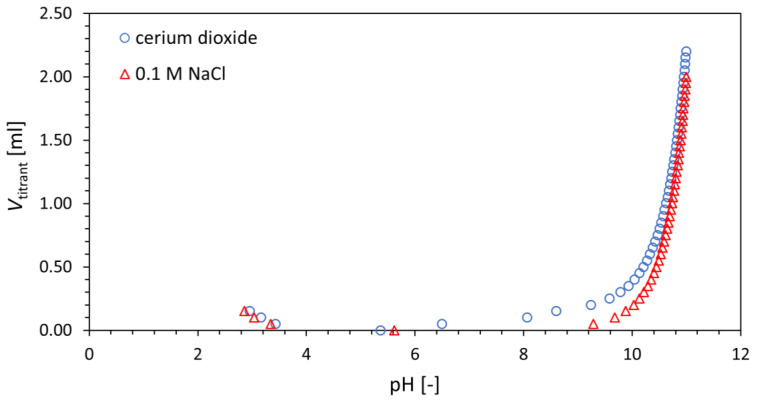
Experimental dependence of titrant volume (either 1 M HCl for acidic part or 0.1 M NaOH for basic part) on pH for tested material and blank titration curve of 0.1 M NaCl.

Titration curve of prepared cerium dioxide is depicted in Figure 5 as a dependence of titrant volume on pH value. The surface area of the prepared material was determined to be 48,760 m^2^/kg. Three mathematical surface complexation models, CCM, DLM, and CEM, were applied to these experimental data. The values of protonation and ion-exchange constants *K*_1_, *K*_2_, and *K*_ex_ as well as total concentration of surface groups ∑SOH and ∑X derived for each model, together with the WSOS/DF of each model, are summarized in Table 1.

As can be seen, all tested models meet the criterion of goodness-of-fit. However, the CEM model seems to be the most suitable, as the values of constants and total concentrations obtained from this model seem to reflect the real system in the best way. Moreover, the values of uncertainty for all constants seem to be reasonably low for this model.

This conclusion can be supported by the results of Kukleva et al. [33], where another oxidic material, titanium dioxide, was evaluated, similar values of total concentrations were gained, and the CEM model was also the model of choice.

**Figure 6:**
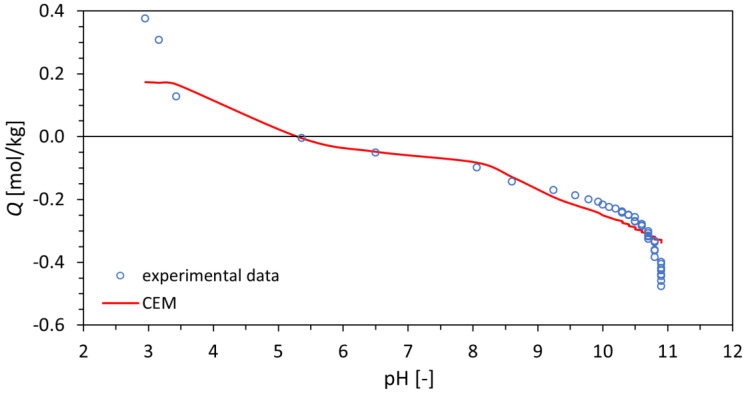
Dependence of total surface charge density *Q* on pH calculated from the titrant consumption as in [32] for experimental data and calculated titration curve using the CEM model.

**Figure 7:**
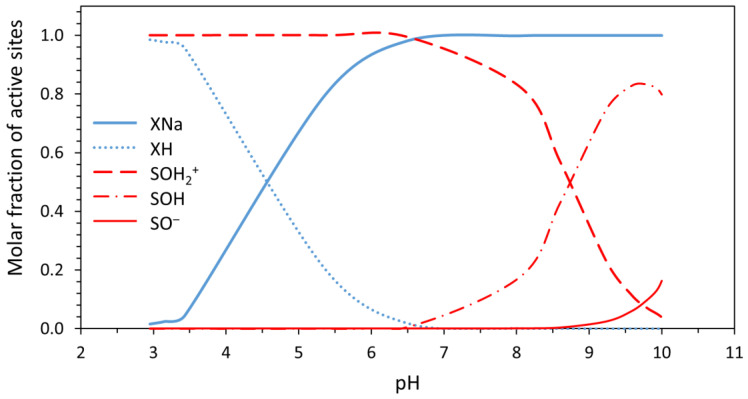
Dependence of molar fraction of active sites forms on pH calculated using the CEM model.

The experimental data and titration curve calculated using the CEM model are depicted as a dependence of total surface charge density on pH in Figure 6. The dependence of the molar fraction of individual forms of sites on pH is depicted in Figure 7.

Comparing these results with the work of Vlasova and Markitan [34], where surface characterization of cerium dioxide using the analogous method of potentiometric titration was used, the importance of the preparation method influencing the size, specific surface area, and crystallographic structure can be seen. In the mentioned work, the isoelectric point of cerium dioxide was found to be almost 1 pH point higher than in the case of our material (see Figure 6), so it excludes the possibility of gaining the quantities in Table 1 characterizing the CCM, DLM, and CEM surface complexation models.

Dependences depicted in Figure 6 and Figure 7 show that especially in the acidic range of pH (up to 5.5), negative surface charge (see [X^−^] ≅ [XNa] layer sites) of the tested cerium dioxide predominates in the system. The same phenomenon was observed in the case of TiO_2_, studied in [33]. The similarity with this material is also observed in the total concentrations of active sites. The total concentration of edge sites ∑SOH for titanium dioxide was found to be 0.20 ± 0.01 mol/kg [33], which is comparable with 0.177 ± 0.003 mol/kg for cerium dioxide. This predicts a similar sorption capacity for these two materials. On the other hand, in the case of cerium dioxide, the predominance of the SOH_2_^+^ form of edge sites in a broad range of pH (approx. 3–8.5) can be seen in Figure 7.

These findings indicate the possibility of anionic species sorption in a wide range of pH, which is applied, for example, in ^68^Ge/^68^Ga radionuclide generators for the sorption of ^68^Ge [5,13]. It could make cerium dioxide preferable as a stationary phase in such generators compared to commercially used titanium dioxide, which does not show this kind of predominance of positive SOH_2_^+^ form in such a broad range of pH [33]. Such a wide range of SOH_2_^+^ predominance can lead to use of a lower concentration of hydrochloric acid utilized for elution of these generators, as already tested in [13], where the ^68^Ge/^68^Ga radionuclide generator using cerium dioxide was eluted by 0.01 M HCl in comparison with 0.1 M HCl in the commercial generator containing titanium dioxide [5,6,11].

The increasing concentration of X^−^ form of layer sites with increasing pH should allow cationic species sorption, mainly at higher values of pH. This finding is in accordance with the generally known amphoteric character of hydrated oxides [4].

### 3.3. Cerium Dioxide Sorption Capacity for Germanium

**Figure 8:**
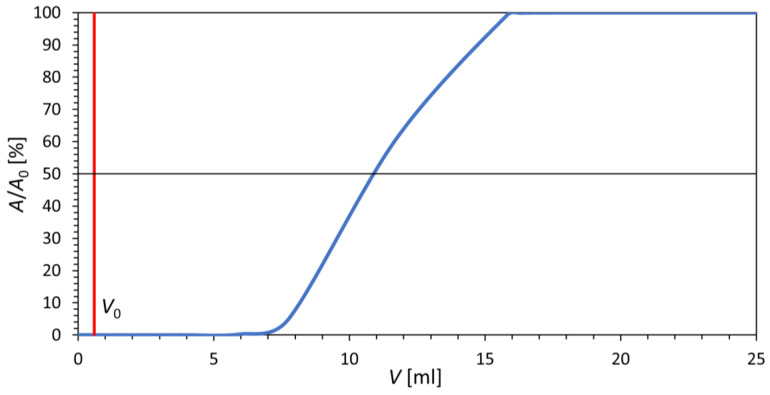
Breakthrough curve of germanium for CeO_2_-PAN composite; BV = 1.25 mL, *V*_0_ = 0.6 mL, *c*_HCl_ = 0.1 mol/L, flow rate = 4 mL/h.

The sorption capacity of the prepared material for germanium was measured in order to revise its applicability in the ^68^Ge/^68^Ga radionuclide generator. The capacity was determined from the breakthrough curve depicted in Figure 8. The measurement required granulation of CeO_2_ into the polymer matrix of PAN as the flow of liquid phase through such a fine powder could not have been provided. The capacity was measured at pH = 1. For the prepared column with a bed volume of 1.25 mL, the capacity was determined to be 2.53 mg of germanium. This means that the capacity of 1 mL of wet composite is 2.02 mg of germanium and the capacity of 1 g of prepared CeO_2_ is 9.11 mg of germanium.

Taking into consideration the use of ^68^Ge stock solution provided by Eckert & Ziegler containing 14.8 MBq ^68^Ge and 10 μg of germanium carrier per 1 mL, the capacity of 1 g of prepared CeO_2_ is approximately 13.4 GBq ^68^Ge and the capacity of 1 mL of wet composite is approximately 3 GBq ^68^Ge. It would allow the preparation of a typical commercial generator.

In [13], the capacity of the prepared CeO_2_-PAN composite was 40 mg of germanium for pH = 3. It can be assumed that the lower the acid concentration for the operation of a potential generator, the greater the capacity reached.

## 4. Conclusions

In this study, cerium dioxide was prepared, characterized, and tested as a means of separating medicinal radionuclides. It was concluded that the chosen synthesis method, calcination of ceric nitrate, is fast, simple, and suitable for preparation of this ion-exchanger. This method provides a crystalline material of great purity with a wide range of particle sizes, from 1 μm to around 10 nm.

The study of surface functional groups and active sites using potentiometric titration and mathematical modeling showed that this material should possess amphoteric properties as anticipated and could be suitable for the separation of ^68^Ge and ^68^Ga in an acidic medium. In comparison with titanium dioxide, the pH range of positive charge predominance is wider, suggesting that the separation of the mentioned radionuclides could be manageable at lower concentrations of acid than in the case of titanium dioxide, which would make cerium dioxide a favorable material. The applicability of the prepared material to a potential ^68^Ge/^68^Ga radionuclide generator was confirmed by the measurement of the sorption capacity for germanium.

The optimal sorption conditions and kinetic parameters of sorption will be further studied in batch, kinetic, and column sorption experiments.

## Figures and Tables

**Table 1:** The total concentration of edge (∑SOH) and layer (∑X) sites, protonation (*K*_1_, *K*_2_) and ion exchange (*K*_ex_) constants, and values of WSOS/DF for the tested models.

Model	∑SOH [mol/kg]	∑X [mol/kg]	*K*_1_ [L/mol]	*K*_2_ [L/mol]	*K*_ex_ [-]	WSOS/DF
CCM	5.10 ± 0.28	0.292 ± 0.003	(2.14 ± 3.45) × 10^16^	(5.14 ± 0.23) × 10^8^	67.00 ± 6.62	7.05
DLM	(4.44 ± 3.12) × 10^3^	0.063 ± 0.001	(1.34 ± 0.73) × 10^12^	45.9 ± 25.1	(1.55 ± 0.08) × 10^4^	1.80
CEM	0.177 ± 0.003	0.228 ± 0.003	(6.90 ± 0.45) × 10^10^	(6.79 ± 0.98) × 10^8^	(5.86 ± 0.36 × 10^3^	3.64

## Data Availability

The data presented in this study are available in this article. Raw data are available on request. The theoretical background for the modeling and evaluation of titration data is available in reference [32].

## Supplementary Materials

The following supporting information can be downloaded at: https://www.mdpi.com/article/10.3390/ma16051758/s1: Figure S1: Intensity-weighted hydrodynamic radius distribution of cerium dioxide, repeated measurements; Figure S2: Volume distribution of cerium dioxide particle size.
