# Supplementary material for: Preparation and Surface Characterization of Cerium Dioxide for Separation of 68Ge/68Ga and Other Medicinal Radionuclides
Source: Materials (Basel). 2023 Feb 21;16(5):1758. doi: 10.3390/ma16051758 (PMC10004219; doi:10.3390/ma16051758)
Supplement: Supplementary file 1 [file materials-16-01758-s001.zip › materials-2217938-supplementary.pdf]

Preparation and surface characterization of cerium dioxide for separation of $^{68}Ge/^{68}Ga$ and other medicinal radionuclides

Kateřina Ondrák Fialová [1*], Kryštof Adámek [1], Martin Vlk [1], Barbora Drtinová [1], Karel Štamberg [1], Ferdinand Šebesta [1], Miroslav Šlouf [2] and Ján Kozempel [1]

[1] Faculty of Nuclear Sciences and Physical Engineering, Czech Technical University in Prague, Břehová 7, 115 19 Prague, Czech Republic
[2] Institute of Macromolecular Chemistry, Czech Academy of Sciences, Heyrovského náměstí 2, 162 06 Prague, Czech Republic
* Correspondence: katerina.fialova@fjfi.cvut.cz; Tel.: +420 728 569 532

Supplementary Information

Dynamic light scattering analysis

For dynamic light scattering analysis Zetasizer NanoZS instrument, model ZEN3600 (Malvern Instruments) was used and the intensity-weighted hydrodynamic radius, R_H, scattering intensity, and zeta potential, ζ, were measured. Repeated measurements at 25°C with scattering angle 173° were carried out. The concentration of cerium dioxide was 0.1 mg/ml in ultrapure water.

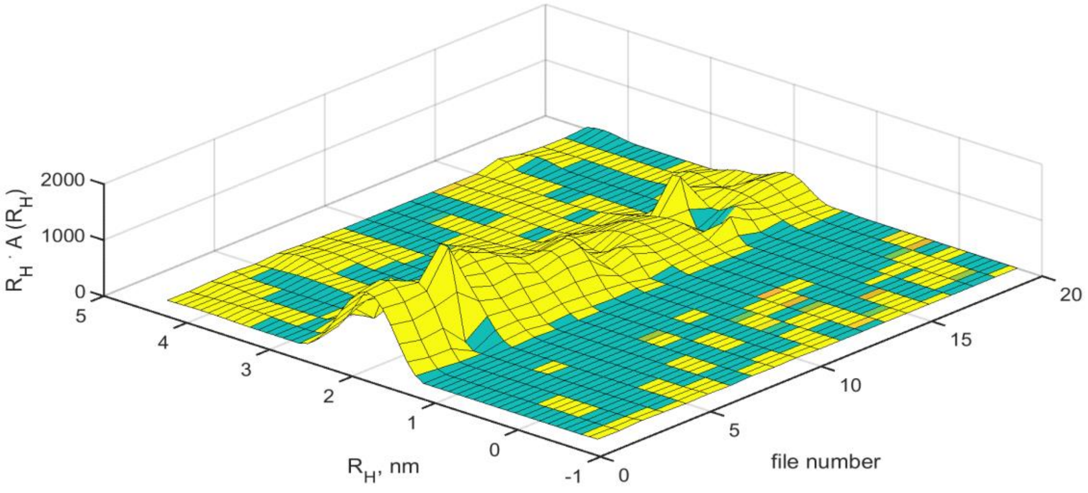

Figure S1: Intensity-weighted hydrodynamic radius distribution, repeated measurements

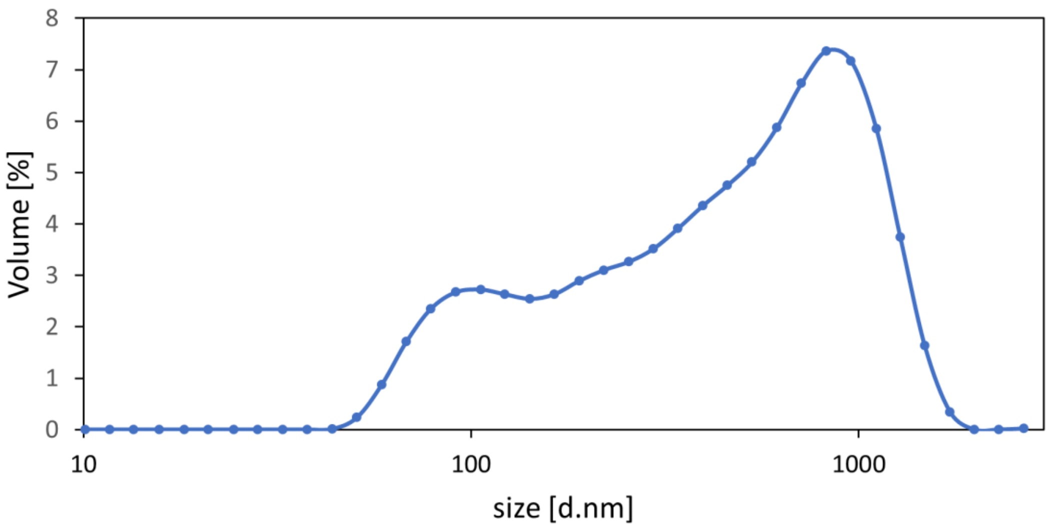

Figure S2: Volume distribution of cerium dioxide particle size
